# Recent Advances in Learning Theory

**DOI:** 10.1155/2015/395948

**Published:** 2015-11-16

**Authors:** Weihui Dai, Wlodzislaw Duch, Abdul Hanan Abdullah, Dongrong Xu, Ye-Sho Chen

**Affiliations:** ^1^School of Management, Fudan University, Shanghai 200433, China; ^2^Department of Informatics, Nicolaus Copernicus University, Toruń, Poland; ^3^Faculty of Computing, Universiti Teknologi Malaysia, Johor, Malaysia; ^4^Psychiatry Department, New York State Psychiatric Institute, Columbia University, New York, NY 10032, USA; ^5^E. J. Ourso College of Business, Louisiana State University, Baton Rouge, LA 70803, USA

## 1. Introduction

In the era facilitated by the Internet of Things, ubiquitous communications as well as cloud services, sensing means, and human-computer interfaces are becoming all-pervasive and online. This makes it more possible for us than ever before to study engineering problems, human activities, and social behaviors through machine learning analysis of the big data produced in the ubiquitous environment. Looking at the recent history and new trends, machine learning has made attractive progress in wide areas of applications, from natural language to nonverbal communication, from engineering application to humanities, arts, and social studies, and from the real world to cyber space.

In 1997, Dietterich summarized the development of machine learning in four directions: ensembles of classifiers, methods for scaling up supervised learning algorithm, reinforcement learning, and learning of complex stochastic models [1]. Latterly, Duch addressed machine learning as the foundations of computational intelligence comprehensively [2]. Since the beginning of the 21st century, research in machine learning has made progress in all of the four directions and has become focused on new challenges in learning from big data that cover a variety of application areas. One barrier that needs to be broken through is how to avoid the “curse of dimensionality” and ensure the generalization ability in the learning process. Owing to the efforts such as work of Koller and Friedman on Probabilistic Graphical Models [3] and the Compressive Sensing (CS) theory [4], the tentative path has been lightened. As the forethought by Wang [5], “predicting” the changes based on generalization ability and “describing” the knowledge discovered from huge data will be the two major tasks of machine learning in the future.

In today's society, machine learning has been in an extensive demand in the areas associated with human's psychology and behaviors, such as ubiquitous learning, e-commerce, online customer service, behavioral finance analysis, and government emergency management. We believe that machine learning could be the most promising, sometimes even the only, way to accomplish the complex computation on human psychology and behaviors in the ubiquitous environment.

However, in doing so, the machine learning research needs to pay attention to the following new aspects which may be beyond the ability of computer science and technology and requires more novel interdisciplinary ideas and methods: (1) a systematic model such as the social neuroscience mechanism [6] which can describe the neural activities and dominant process of human psychology and behaviors and helps the machine to understand its globally structural features and therefore reduce the computational cost by learning from the limited samples of a big data set, (2) the comprehensive context awareness in physical, cyber, and psychosocial spaces, as well as the information fusion processing and computing ability, which has been called Cyber Psychosocial and Physical (CPP) Computation by Dai [7], and (3) smart learning that enables the machine to cope with both rational intelligence and emotional intelligence in the learning process.

The aim of this special issue is to bring together researchers working in different areas for exchanging and sharing with each other their progress in the new tendency of modern learning theory and applications. We were very pleased to see various new research ideas and many innovative contributions in the submitted manuscripts, which cover a wide range of recent advances in learning theory, techniques, and applications. What follows is a brief editorial review of the published papers in this special issue from four perspectives: Neuroscience in Learning Theory, Machine Learning in Psychological Computation and Behavior Analysis, Machine Learning in Public Management and Business Service, and Machine Learning in Knowledge Discovery and Human-Computer Engineering.

## 2. Neuroscience in Learning Theory

The paper titled “Neural Cognition and Affective Computing on Cyber Language” by S. Huang et al. analyzed the classification and cognitive characteristics of emotional symbols in cyber languages and put forward a mechanism model to show the dominant neural activities in that cognitive process. Through the comparative study of Chinese, English, and Spanish languages in their expressive patterns of emotions, an intelligent method of machine learning was proposed for affective computing on international cyber languages which can deal with the multisymbol information and mixed emotions in a cyber message and show their dynamic changes according to the characteristics of the neural cognition process.

The paper titled “Neural Basis of Intrinsic Motivation: Evidence from Event-Related Potentials” by J. Jin et al. employed event-related potentials (ERPs) to investigate the neural disparity between an interesting stop-watch (SW) task and a boring watch-stop (WS) task to understand the neural mechanism of intrinsic motivation. Research findings of this paper indicated that intrinsic motivation could be added as a candidate social factor in the construction of a machine learning model, and it provided the new possible indicators as well as the feature parameters for detecting and analyzing human intrinsic motivation based on machine learning technology in a wearable system.

The paper titled “A Neuroeconomics Analysis of Investment Process with Money Flow Information: The Error-Related Negativity” by C. Wang et al. studied the features of event-related potentials (ERPs) in the decision-making process of financial investment on stock market. Experimental results showed that ERN component was sensitive to the evaluation of the risk in the investment decision process and could be regarded as the early warning indicator to show a conflict perception or alert the brain to prepare for potential negative consequences. Findings in this paper have significant implications for exploring the neural cognitive effects and the basis of machine learning from ERP signals in the development of a financial intelligence system.

The paper titled “P300 and Decision Making under Risk and Ambiguity” by L. Wang et al. used ERPs to clarify and extend the current understanding of decision-making under risk and with ambiguity. Research findings pointed out that decision-making with ambiguity occupies larger amount of working memory and recalls more past experience, while decision-making under risk mainly mobilizes attention resources to calculate current information. This paper provided further understanding of brain mechanism in decision-making, which may be helpful in the design of humanoid robots based on machine learning theory.

The paper titled “Explore Awareness of Information Security: Insights from Cognitive Neuromechanism” by D. Han et al. took the online financial payment as a research example and conducted an experimental analysis of electrophysiological signals to study the awareness of information security. Its findings indicated that left hemisphere and beta rhythms of electroencephalogram (EEG) signal are sensitive to the cognitive degree of risks in the awareness of information security, which may be probably considered as the sign to assess people's cognition of potential risks in online financial payment. This paper contributed new knowledge to the understanding of EEG signals in the awareness of information security, which is of significance for the development of machine learning technology for the objective and technological assessment of information security awareness.

## 3. Machine Learning in Psychological Computation and Behavior Analysis

The paper titled “CyberPsychological Computation on Social Community of Ubiquitous Learning” by X. Zhou et al. studied the relationships between the ubiquitous learners' psychological reactions and their behavioral patterns in cyber space and summarized 15 common basic actions of the learners' habitual behaviors for the psychological assessment of their situations in the learning process. A CyberPsychological computation method based on BP-GA neural network was proposed by the authors, which can be used to estimate the learners' psychological states online according to their personalized behavioral patterns. Contributions of this paper provided new progresses in the field of machine learning on Cyber Psychosocial and Physical (CPP) Computation and have important significance for further research in wide application areas.

The paper titled “An Opinion Interactive Model Based on Individual Persuasiveness” by X. Zhou et al. discussed a common phenomenon in social interactive process which is associated with the propagation characteristics of public opinions and attitudes to a social event. Based on the Deffuant Model and its improvements, the authors considered the impacts of individual persuasiveness and conducted an experiment using multiagent simulation to show that the range of common opinion could be predicted when the initial distribution of opinions and persuasiveness are given. The interesting findings in this paper indicated that there would be some underlying rules for machine learning in the behavior analysis of a social interactive process.

In addition, studies of machine learning on affective computing were also reported by S. Huang et al. in the paper titled “Neural Cognition and Affective Computing on Cyber Language” and by S. Gong et al. in the paper titled “Emotion Analysis of Telephone Complaints from Customer Based on Affective Computing.”

## 4. Machine Learning in Public Management and Business Service

The paper titled “Information Dissemination of Public Health Emergency on Social Networks and Intelligent Computation” by H. Hu et al. emphasized that social networks had become the main information dissemination platform of public health emergency and caused high concerns in emergency management. The authors analyzed the complex characteristics of information dissemination in social networks in public health emergency and argued that the existing theoretical tools and modeling methods are not sufficient to accurately describe and predict the information dissemination in social networks. Based on the framework of TDF (Theory-Data-Feedback), a new intelligent computation method was constructed for the ACP (artificial societies, computational experiments, and parallel execution) simulation by this paper and reached highly precise results on the prediction of dynamic dissemination of emergency event's information on social networks.

The paper titled “The Large Scale Machine Learning in an Artificial Society: Prediction of the Ebola Outbreak in Beijing” by P. Zhang et al. proposed a new method for predicting the disease propagation based on artificial society. The authors established the virtual society system of “Artificial Beijing” which contained 19.6 millions of individuals and 8 millions of buildings in correspondence with the actual distributions of geodemographics, infrastructure, and social roles in Beijing. Through a large scale machine learning of individuals' behaviors, the propagation process of Ebola virus disease and its corresponding interventions for public health emergency management are well simulated and accurately predicted based on “Artificial Beijing.”

The paper titled “Emotion Analysis of Telephone Complaints from Customer Based on Affective Computing” by S. Gong et al. studied the characteristics of telephone complaint speeches and proposed an analysis method based on affective computing technology, which can recognize the dynamic changes of customer emotions from the conversations between the service staff and the customer. Experimental results showed that this method is effective and could reach high recognition rates of happy and angry states. It has been successfully applied to the operation quality and service administration in telecom and Internet service company.

## 5. Machine Learning in Knowledge Discovery and Human-Computer Engineering

The paper titled “Exploiting Language Models to Classify Events from Twitter” by D.-T. Vo et al. studied the issue of event classification in Twitter based on the models of LDA-SP and ConceptNet. The authors explored a new method to compute tweets' similarity according to their features, including common term words and the relationships among their distinguishing term words, by the kNN classifier and experiments on Edinburgh Twitter Corpus. This paper gives a logical and comprehensive insight into the research issue, presenting an effective method which exhibits the valuable exploration by adopting the language model to improve the classification performance as shown in the experiments.

The paper titled “Design of Automatic Extraction Algorithm of Knowledge Points for MOOCs” by H. Chen et al. studied knowledge discovery and knowledge sharing in MOOCs (Massive Online Open Courses). The authors designed an automatic extracting course knowledge points (AECKP) algorithm for building the course ontology and employed the Vector Space Model (VSM) to calculate the similarity between knowledge points for the optimization of knowledge extraction. Experimental results showed that the proposed approach could achieve satisfactory results.

The paper titled “Intelligent Context-Aware and Adaptive Interface for Mobile LBS” by J. Feng and Y. Liu discussed an interesting issue about the intelligent interface for mobile LBS (Location Based Services). Through penetrating analysis of the requirements on the interface's design, the authors proposed a context-aware adaptive model for LBS interface along with the framework for its application and described the adaptive process of dynamic interaction between users and the interface. A standard usability evaluation test was conducted to show the performance improvements by the proposed model. Research work of this paper provided a valuable reference for the design of adaptive interface based on context awareness.

Finally, the paper titled “An Efficient Robust Eye Localization by Learning the Convolution Distribution Using Eye Template” by X. Li et al. presented a novel eye localization approach which explored only one-layer convolution map by eye template using a backpropagation (BP) network. Based on the extracted convolution distributed features and a multieye template set which considered the utilizations of both the global information and the local geometric features, the proposed method could obtain similar best results with greatly reduced training time and high prediction speed and provided a better comprehension compared with existing methods.
